# Circulating Endothelial Progenitor Cells Are Preserved in Female Mice Exposed to Ambient Fine Particulate Matter Independent of Estrogen

**DOI:** 10.3390/ijms22137200

**Published:** 2021-07-04

**Authors:** Xuanyou Liu, Yichao Xiao, Qingyi Zhu, Yuqi Cui, Hong Hao, Meifang Wang, Peter J. Cowan, Ronald J. Korthuis, Guangfu Li, Qinghua Sun, Zhenguo Liu

**Affiliations:** 1 Center for Precision Medicine, Department of Medicine, Division of Cardiovascular Medicine, School of Medicine, University of Missouri, Columbia, MO 65212, USA; liuxua@health.missouri.edu (X.L.); yichaoxiao@csu.edu.cn (Y.X.); zhuqingyi@csu.edu.cn (Q.Z.); cuiyu@health.missouri.edu (Y.C.); haoho@health.missouri.edu (H.H.); wangmei@health.missouri.edu (M.W.); 2 Department of Medical Pharmacology and Physiology, School of Medicine, University of Missouri, Columbia, MO 65212, USA; korthuisr@health.missouri.edu; 3 Immunology Research Centre, Department of Medicine, St. Vincent’s Hospital, University of Melbourne, Melbourne 3065, Australia; peter.cowan@svha.org.au; 4 Dalton Cardiovascular Research Center, University of Missouri, Columbia, MO 65211, USA; 5 Department of Surgery and Department of Molecular Microbiology and Immunology, School of Medicine, University of Missouri, Columbia, MO 65212, USA; liguan@health.missouri.edu; 6 College of Public Health, Ohio State University, Columbus, OH 43210, USA; Sun.224@osu.edu

**Keywords:** PM exposure, endothelial progenitor cell, ROS, oxidative stress, estrogen

## Abstract

Males have a higher risk for cardiovascular diseases (CVDs) than females. Ambient fine particulate matter (PM) exposure increases CVD risk with increased reactive oxygen species (ROS) production and oxidative stress. Endothelial progenitor cells (EPCs) are important to vascular structure and function and can contribute to the development of CVDs. The aims of the present study were to determine if sex differences exist in the effect of PM exposure on circulating EPCs in mice and, if so, whether oxidative stress plays a role. Male and female C57BL/6 mice (8–10 weeks old) were exposed to PM or a vehicle control for six weeks. ELISA analysis showed that PM exposure substantially increased the serum levels of IL-6 and IL-1β in both males and females, but the concentrations were significantly higher in males. PM exposure only increased the serum levels of TNF-α in males. Flow cytometry analysis demonstrated that ROS production was significantly increased by PM treatment in males but not in females. Similarly, the level of circulating EPCs (CD34^+^/CD133^+^ and Sca-1^+^/Flk-1^+^) was significantly decreased by PM treatment in males but not in females. Antioxidants N-acetylcysteine (NAC) effectively prevented PM exposure-induced ROS and inflammatory cytokine production and restored circulating EPC levels in male mice. In sharp contrast, circulating EPC levels remained unchanged in female mice with PM exposure, an effect that was not altered by ovariectomy. In conclusion, PM exposure selectively decreased the circulating EPC population in male mice via increased oxidative stress without a significant impact on circulating EPCs in females independent of estrogen.

## 1. Introduction

Ambient fine particulate matter (PM) is a key component of air pollution, which is associated with significant morbidity and mortality [1]. A recent Global Burden of Disease Study revealed that PM was responsible for 4.2 million deaths in 2015, an increase of 7.8% from 2005 [2]. The majority of mortality following PM exposure is related to cardiovascular diseases (CVDs), including high blood pressure, arrhythmias, atherosclerosis, myocardial infarction, and heart failure [3]. PM is a mixture of various particles from natural sources and human activities. Fine particles with an aerodynamic diameter of 2.5 μm (PM_2.5_) or less may penetrate the lung alveoli and enter the bloodstream, and are largely responsible for cardiovascular morbidity and mortality associated with PM exposure [4,5].

CVDs remain the leading cause of morbidity and mortality in developed countries [6,7]. Endothelial cell (EC) dysfunction is associated with the development and progression of CVDs, including high blood pressure, arrhythmias, atherosclerosis, stroke, myocardial infarction, and heart failure [8,9]. Endothelial progenitor cells (EPCs) play a critical role in maintaining the structural and functional integrity of the cardiovascular system [10,11]. A decreased number and/or function of EPCs are associated with the development and progression of CVDs and their outcomes [12,13]. Previous studies have shown that PM exposure significantly suppresses the number and function of EPCs in animals and human subjects [14,15].

Excessive reactive oxygen species (ROS) production and related oxidative stress contribute significantly to the development and progression of a variety of cardiovascular diseases, such as atherosclerosis, hypertension, cardiomyopathy, and myocardial infarction [16,17]. Air pollution and PM exposure increase ROS production and oxidative stress [18,19]. Our previous studies demonstrated that PM exposure dramatically suppresses the number of circulating EPCs through excessive ROS production in male mice [14]. However, it is unclear whether PM exposure also decreases the population of circulating EPCs in female mice, and if so, would occur by a similar mechanism. It is also possible that PM exposure may exert little effect on circulating EPC levels, perhaps owing to protective actions of estrogens or some other mechanism.

There are substantial sex differences in various CVDs, including (but not limited to) myocardial infarction, heart failure, hypertension, shock, and cardiac hypertrophy [20]. However, the mechanisms for sex differences in CVDs have not been defined. The effects of sex hormones such as estrogen are one of the biggest differences between males and females, which could contribute to sex differences. The objectives of the present study were to: (1) Determine if sex differences alter circulating EPCs levels in mice following PM exposure; (2) define the role of ROS production and oxidative stress in mediating the effect of PM exposure on the sex differences in circulating EPCs levels; and (3) evaluate the effect of estrogen on ROS production and circulating EPCs levels in mice with PM exposure by comparing responses in female mice with and without ovariectomy. To achieve these objectives, both male and female mice were used for the study. Reductions in oxidative stress was achieved using a FDA-approved antioxidant N-acetylcysteine (NAC). The antioxidant property of NAC is attributed to its ability to function as a reduced glutathione (GSH) precursor, which is a direct antioxidant that also acts as a substrate for a variety of antioxidant enzymes [21].

## 2. Results

### 2.1. PM Exposure Decreased Circulating EPCs with an Increased Apoptosis Rate in Male Mice but Not in Female Mice

**Figure 1:**
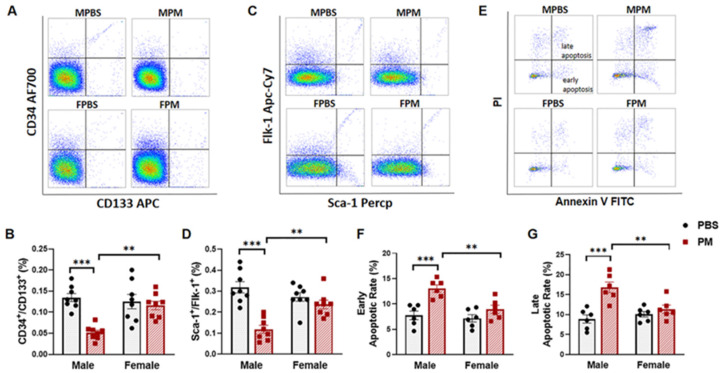
PM exposure selectively decreased circulating EPC levels with an increased apoptosis rate in male mice. (**A**,**C**) White blood cells were stained with CD133 APC and CD34 AF700 or Sca-1 Percp and Flk-1 Apc-cy7 antibodies for flow-cytometric analysis of circulating EPCs (CD34^+^/CD133^+^) or (Sca-1^+^/Flk-1^+^), with summary data (**B**,**D**) showing that decreased levels of circulating EPCs were only observed in WT male mice with PM exposure (*n* = 8). (**E**) Annexin V and PI were used to incubate the blood cells for apoptosis analysis, which gated from EPCs (CD34^+^/CD133^+^), with summary data showing that both early (Annexin V^+^/PI^-^) (**F**) and late (Annexin V^+^/PI ^+^) (**G**) apoptotic rates of EPCs in male mice with PM exposure were significantly increased as compared to the PBS control group and female mice (*n* = 6). MPBS: male mice with PBS treatment; MPM: male mice with PM exposure; FPBS: female mice with PBS treatment; FPM: female mice with PM exposure. ** *p* < 0.01 and *** *p* < 0.001.

Levels of circulating EPCs were analyzed in mice after six weeks of PM exposure using flow cytometry. As expected, the percentage of blood cells double-positive for CD34^+^/CD133^+^ and Flk-1^+^/Sca-1^+^ (EPCs) was significantly decreased in male mice with PM exposure, as compared to the control group. However, no significant change in either cell population was observed in female mice with PM exposure (Figure 1A–D). Flow cytometry analysis also showed that the apoptosis rate in these cell populations was significantly increased in male mice with PM exposure, but not in female mice with PM exposure (Figure 1E–G).

### 2.2. PM Exposure Significantly Increased Serum Pro-Inflammatory Cytokines and Intracellular ROS Levels

**Figure 2:**
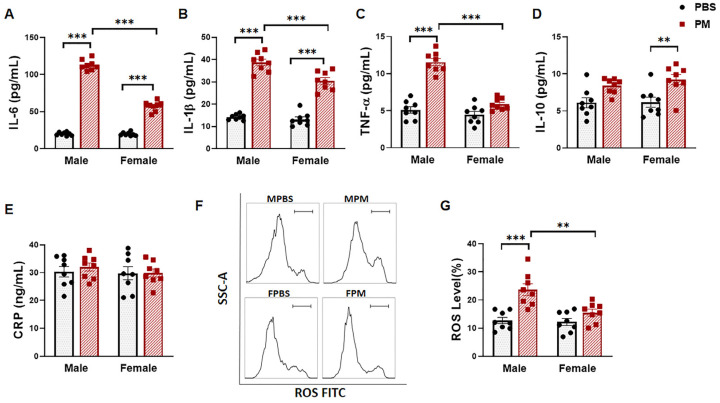
PM exposure increased serum cytokines and ROS production. (**A**–**C**) Serum pro-inflammatory cytokines IL-6 (**A**) and IL-1β (**B**) were significantly increased in both male and female mice with PM exposure compared to the PBS control, while the serum levels of pro-inflammatory cytokines in male mice were significantly higher than those in female mice. (**C**) pro-inflammatory TNF-α was significantly increased in male mice with PM exposure, but not in female mice. (**D**) Anti-inflammatory cytokine IL-10 was increased in female mice with PM exposure compared to the PBS control. (**E**) No significant change was observed in the level of serum CRP. (**F**) Flow cytometry analysis was used to detect the ROS in blood monocytes, with summary data (**G**) showing a significant increase in ROS production in male mice with PM exposure compared to the control group and female mice with PM exposure. MPBS: male mice with PBS treatment (*n* = 8); MPM: male mice with PM exposure (*n* = 8); FPBS: female mice with PBS treatment (*n* = 8); FPM: female mice with PM exposure (*n* = 8). ** *p* < 0.01 and *** *p* < 0.001.

The levels of serum pro-inflammatory cytokines IL-6, IL-1β, and TNF-α, anti-inflammatory cytokine IL-10, and the inflammatory biomarker CRP were measured using ELISA. PM exposure substantially increased the serum levels of IL-6 and IL-1β, both in male and female mice. However, the levels induced by PM exposure were significantly higher in males than in females by 49.0% and 21.3% for IL-6 and IL-1β respectively (Figure 2A,B). TNF-α was only increased in male mice with PM exposure but not in female mice (Figure 2C). On the contrary, IL-10 was significantly increased in females with PM exposure, while no significant change was observed in males (Figure 2D). However, there was no significant change in the serum CRP level in either males or females with PM exposure (Figure 2E). Similarly, the blood cell intracellular ROS level was significantly increased in males with PM exposure, but not in females (Figure 2F,G).

### 2.3. Antioxidant NAC Treatment Prevented PM Exposure-Induced Production of Inflammatory Cytokines and Reduction of Circulating EPCs in Male Mice

**Figure 3:**
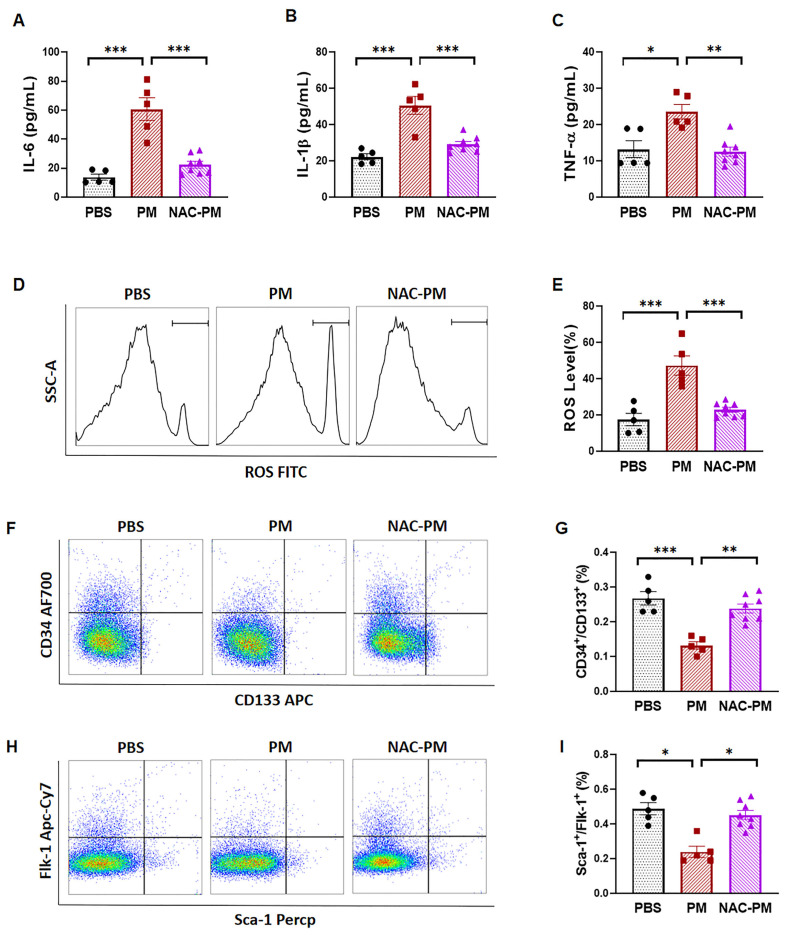
NAC preserved the circulating EPCs in male mice with PM exposure. (**A–C**) The elevated serum levels of pro-inflammatory cytokines IL-6 (**A**), IL-1β (**B**), and TNF-α (**C**) induced by PM were effectively prevented by NAC treatment. (**D**) Increased ROS production exposed to PM was also blocked by NAC treatment. (**E**) Quantification data from (D). (**F–I**), Flow cytometry analysis for circulating EPCs (CD34^+^/CD133^+^) or (Sca-1^+^/Flk-1^+^), with summary data (**G,I**) showing that the PM exposure-induced decrease in the circulating EPC level in male mice was restored after NAC treatment. PBS: male mice with PBS treatment (*n* = 5); PM: male mice with PM exposure (*n* = 5); NAC-PM: male mice with PM exposure and NAC treatment (*n* = 8). * *p* < 0.05, ** *p* < 0.01, and *** *p* < 0.001.

To determine whether ROS production is critical to the PM exposure-induced production of pro-inflammatory cytokines, as well as a decrease in circulating EPC populations, male mice were treated with antioxidant NAC to inhibit oxidative stress. As expected, no significant increases in the serum levels of IL-6, IL-1β, or TNF-α were observed in mice co-treated with PM and NAC (Figure 3A–C). In association with the decreased level of pro-inflammatory cytokines, the intracellular ROS production was completely blocked by NAC treatment with PM exposure compared to the controls (Figure 3D,E). Next, we examined whether inhibition of ROS production could maintain the EPC population during PM exposure. As shown in Figure 3F,H, the decreased EPC population by PM was completely restored by NAC treatment. All in all, the present study suggests that PM exposure significantly decreases the circulating EPC population in male mice with increased ROS and inflammatory cytokines, but not in female mice. NAC decreased ROS production and restored the population of EPCs in male mice exposed to PM.

### 2.4. Ovariectomy Had no Effect on the Circulating EPC Population in Female Mice with PM Exposure

**Figure 4:**
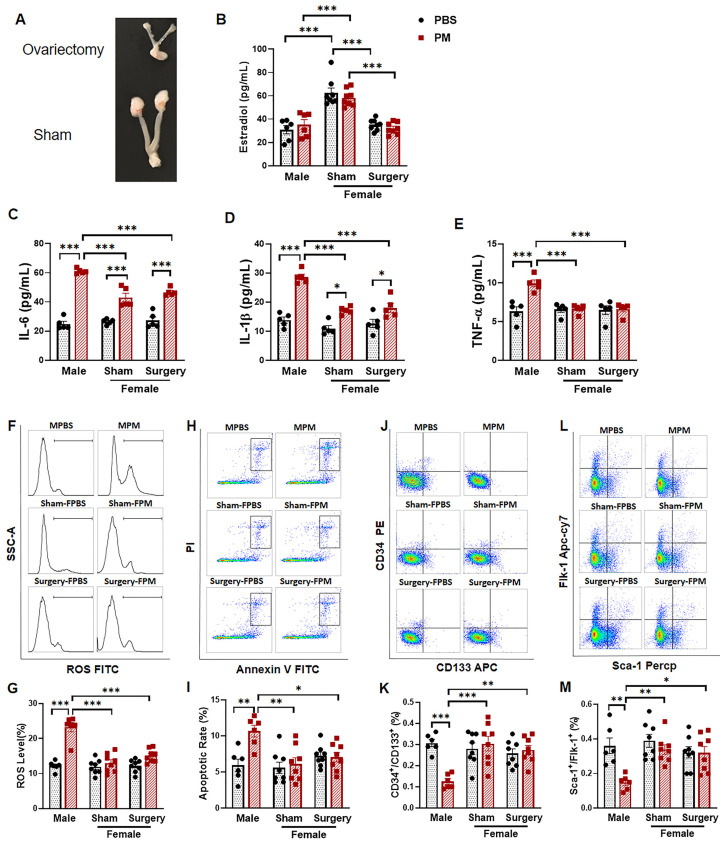
The protective effect on circulating EPCs in female mice with PM exposure was independent of estrogen. (**A**) The size of the uterus and ovary tissue from female mice with ovariectomy was much smaller than that from female mice with sham operation. (**B**) Serum level of estradiol was significantly decreased in female mice with ovariectomy to a level similar to male mice. (**C,D**) Pro-inflammatory cytokines IL-6 (C) and IL-1β (D) in male mice with PM exposure were significantly higher than female mice with or without ovariectomy. (**E**) Elevated serum levels of TNF-α induced by PM exposure were only observed in male mice, not in female mice with or without ovariectomy. (**F**) Flow cytometry analysis was used to detect the ROS in blood monocytes, with summary data (**G**) showing that a significant increase in ROS production was observed in male mice with PM exposure, but not in female mice with or without ovariectomy after PM exposure. (**H**) Blood cells were incubated with annexin V and PI for apoptosis analysis, with summary data (**I**) showing a significant increase in the apoptotic rate only in male mice with PM exposure, not in female mice with or without ovariectomy (**G**). (**J,L**) Flow cytometry analysis for circulating EPC (CD34^+^/CD133^+^) or (Sca-1^+^/Flk-1^+^), with summary data (**K**,**M**) showing a significantly decreased circulating EPC population in male mice after PM exposure, not in female mice with or without ovariectomy. MPBS: male mice with PBS treatment (*n* = 6); MPM: male mice with PM exposure (*n* = 6); Sham-FPBS: female mice with intact ovary and PBS treatment (*n* = 8); Sham-FPM: female mice with intact ovary and PM exposure (*n* = 8); Surgery-FPBS: female mice with ovariectomy and PBS treatment (*n* = 8); Surgery-FPM: female mice with ovariectomy and PM exposure (*n* = 8). (C–E) *n* = 5 for each group. * *p* < 0.05, ** *p* < 0.01, and *** *p*< 0.001.

To determine if the relative resistance of female mice to PM exposure-induced effects on circulating EPCs was due to the female sex hormone estrogen, the experiments were repeated using female mice with and without ovariectomy. As expected, the uterus of female mice following ovariectomy was much smaller than that of the sham control mice (Figure 4A), and the serum estradiol level in female mice with ovariectomy was significantly decreased to the level of male mice six weeks after ovariectomy surgery (Figure 4B), confirming successful creation of the ovariectomy model.

The serum levels of pro-inflammatory cytokines IL-6 and IL-1β were significantly increased in both males and females after PM exposure, but the concentrations were significantly higher in males (Figure 4C,D). There were no differences in IL-6 or IL-1β production in female mice with or without ovariectomy after PM exposure. TNF-α was significantly increased only in male mice following PM exposure—effects not observed in intact female mice or following ovariectomy (Figure 4E). Intracellular ROS was increased significantly in male mice with PM exposure, while there was no significant change in ROS production in female mice with or without ovariectomy after PM exposure (Figure 4F,G). The apoptosis rate of EPCs was significantly higher in male mice after PM treatment compared to the PBS control, while no significant change was observed in females with or without ovariectomy after PM exposure (Figure 4H,I). Similarly, the PM exposure-induced decrease in circulating EPC populations was apparent only in male mice, not in females with or without ovariectomy (Figure 4J–M).

## 3. Discussion

Significant differences between males and females are observed in the presentation, severity, and progression of a variety of diseases, especially cardiovascular diseases. PM_2.5_ exposure is associated with an increased risk for CVDs. However, it is unclear if there is a significant difference in cardiovascular injury in response to PM exposure between males and females. In the present study, we used a mouse model to demonstrate that PM exposure decreases the circulating EPC population in association with increased pro-inflammatory cytokines and intracellular ROS production in males but not in females. NAC treatment effectively attenuated intracellular ROS production and serum IL-6, IL-1β, and TNF-α levels, and restored the circulating EPC population in male mice with PM exposure. We further demonstrated that ovariectomized female mice exhibited the same level of serum estrogen as male mice, showing similar responses to PM exposure in the production of pre-inflammatory cytokines and ROS, as well as in circulating EPCs as female mice without ovariectomy. Taken together, these data suggest that PM exposure selectively decreases the population of circulating EPCs in male mice through increased oxidative stress by a mechanism independent of estrogen.

Air pollution and PM exposure have been reported to significantly impair the number and function of EPCs. After PM or nickel nanoparticle exposure, the number of EPCs (CD34^+^/CD31^+^/CD45^+^/CD133^+^, CD34^+^/VEGF-R2^+^/CD11b^−^, CD34^+^/KDR^+^, CD34^+^/KDR^+^/CD45^−^, or CD34^+^/KDR^+^/CD133^+^) in the circulation has been shown to be significantly decreased in both human and murine studies [15,22,23]. The mechanisms for the decreased number and function of EPCs due to PM or other hazardous particle exposure are complex and multifactorial. Exposure to PM impairs VEGF-mediated signaling to reduce the mobilization of bone marrow-derived EPCs to the circulation [24]. It has also been shown that diesel exhaust particles (DEPs) can reduce the number and function of EPCs with impaired migratory capacity and neoangiogenesis through the disruption of stromal cell-derived factor (SDF)-1 signaling in mice. In addition, DEP promotes ROS generation and the apoptosis of cultured human EPCs and impairs their migration [25]. ROS is known to interact with many molecules to increase the level of cellular oxidative stress. PM exposure is known to increase oxidative stress systemically, and is associated with diseases of various organ systems [26,27]. PM_2.5_ inhalation induces pulmonary oxidative stress and leads to VEGF resistance and EPC dysfunction [18].

There are extensive interactions between inflammatory cytokines and ROS formation. On the one hand, cytokines trigger ROS formation, while on the other hand, ROS can stimulate the production of pro-inflammatory cytokines by activating transcription factor nuclear factor-κB (NF-κB) [28]. Hydrogen peroxide (H_2_O_2_) is a major source of ROS that plays an important role in redox regulation. Transmembrane NADPH oxidases (NOXs) are the main endogenous enzymatic sources of H_2_O_2_, which are generated in response to several cytokines, then regulates ROS production [29]. TNF can activate NOX1 or NOX2 complexes, converting extracellular O_2_ into O_2_^-^ [30]. O_2_^-^ can be very rapidly converted into H_2_O_2_ by SODs, and H_2_O_2_ in turn can stimulate TNF production through activation of the p38 and JNK signaling pathways [31]. Increased production of ROS and inflammatory cytokines is observed in various diseases, such as diabetic nephropathy, heart failure, and psychosis [32,33,34]. Antioxidant enzymes and antioxidant supplementation have been widely used to provide a protective function for cardiovascular diseases. We previously showed that PM exposure induces ROS production and increases serum levels of TNF-α and IL-1β [14]. We expanded on these previous studies by demonstrating that PM exposure induces the production of inflammatory cytokines TNF-α, IL-1β, and IL-6 and significant oxidative stress in male mice, effects that were effectively prevented after treatment with antioxidant NAC in the present study.

Females are generally better protected than males against cardiovascular diseases due to undefined mechanisms [35,36]. Males appear to develop cardiovascular diseases earlier than females, while cardiovascular disease risk increases for women after menopause. Males tend to have a poorer prognosis for various acute inflammatory diseases with a more pronounced inflammatory responses than females [37,38,39]. Male rats with pulmonary arterial hypertension develop more severe perivascular inflammation in the lungs with fibrotic changes in the vascular wall of small pulmonary arteries and the right ventricle myocardium than females [40]. Indeed, increased inflammatory cytokine expression was noted in male mice after PM exposure in the present study. The mechanism(s) for the differential response in pro-inflammatory cytokine production and ROS formation to PM exposure in male and female mice is unclear at this point. It could be because of the effects of sex hormones and/or their active metabolites [41]. The prevalence of CADs is greater in young women with oophorectomy than those with intact ovaries [42]. Estrogen product 17β-estradiol improves insulin signaling and insulin resistance in aged female hearts [43]. However, the Heart and Estrogen/Progestin Replacement Study (HERS), conducted in postmenopausal women with pre-existing CVDs (44–79 years old), showed that female hormone replacement therapy had no beneficial effect on primary or secondary cardiovascular outcomes [44]. These controversies suggest that other estrogen-independent mechanisms may be involved. Genomic factors and estrogen-independent activation of regulatory T cells (Treg) have been shown to provide protection to females by reducing the inflammatory response and regulating the immune system independent of sex hormones [45,46]. In the present study, no differences in pro-inflammatory cytokine production and ROS formation, or in circulating EPC levels, were observed in female mice with or without ovariectomy after PM exposure, suggesting that the effects of PM exposure on the circulating EPC level were independent of estrogen.

Oxidative stress could lead to the differential expression of certain genes that are involved in inflammatory pathways, and may contribute to sex differences in oxidative stress. Studies have shown that ROS production and oxidative stress are higher in vascular cells from male rats than females [47]. In addition, the levels of some oxidative stress biomarkers are higher in young men than in age-matched women, and females have a greater antioxidant capacity than males [48]. It has also been reported that the SOD activity levels in brain and lung are higher in female mice than in male mice, while no differences are present in the kidneys or heart [49]. Further studies are needed to define the mechanism(s) for the sex differences in pro-inflammatory cytokine production, ROS formation, and circulating EPCs in response to PM exposure.

## 4. Materials and Methods

### 4.1. PM Exposure and Animal Model

All of the animal experiments and protocols were reviewed and approved by the Animal Care Committee of the University of Missouri-Columbia, MO, USA. Wild-type (WT) male and female C57 BL/6 mice (8–12 weeks old) were obtained from Jackson Laboratory (Maine, USA) and used in the present study. PM (Standard Reference Materials 2786) was procured from the National Institute of Standards and Technology (NIST). This PM has a mean aerodynamic diameter of 2.8 µm (particle size characteristics in atmospheric particulate material and similar matrices) with the major components including polycyclic aromatic hydrocarbons (PAHs), nitro-substituted PAHs (nitro-PAHs), polycyclic aromatic hydrocarbons (PAHs), hexabromocyclododecane (HBCD) isomers, polybrominated diphenyl ether (PBDE) congeners, sugars, polychlorinated dibenzo-p-dioxin (PCDD), and dibenzofuran (PCDF) congeners and inorganic constituents [50]. PM particles were dispersed by ultrasonication in endotoxin-free PBS at a concentration of 0.5 µg/μL as described [14]. Mice were anesthetized with 2% isoflurane and treated with 10 µg PM three times per week for six weeks via intranasal instillation, with endotoxin-free PBS as the control. To block ROS formation, mice were pre-treated with NAC (1 mg/mL in their drinking water) for 24 h prior to PM exposure as described [14].

### 4.2. Measurement of Inflammatory Cytokines and Estradiol

Mouse blood samples were obtained after six weeks of PM or vehicle exposure. Blood serum was obtained by centrifuging these blood samples for 20 min at 300 g. The pro-inflammatory cytokines tumor necrosis factor (TNF)-α (Cat#430904), interleukin (IL)-1β (Cat#432604), and IL-6 (Cat#431304), as well as anti-inflammatory cytokine IL-10 (Cat#431414), were measured with an ELISA kit (BioLegend, San Diego, CA, USA). Serum C-reactive protein (CRP, R&D System, Cat#MCRP00), and estradiol (R&D systems, Cat#KGE014) were quantitatively determined using ELISA kits as per the manufacturer’s protocols.

### 4.3. Circulating Endothelial Progenitor Cell Analysis

Blood cells were collected and prepared from blood samples obtained from mice after six weeks of PM or vehicle exposure for EPC analysis following the removal of red blood cells (RBCs) using RBC lysis buffer as described [51]. EPCs are a group of very heterogeneous cell populations with different phenotypes and biological characteristics without unified criteria. However, for comprehensive EPC analysis, a variety of cell surface markers for the identification of circulating EPCs were used as described [15,22], including CD34^+^/Flk-1^+^, Sca-1^+^Flk-1^+^, c-Kit^+^/CD31^+^, and CD34^+^/CD133^+^. Cell populations were carefully compensated (each cell population percentile was further confirmed with single antibody staining and fluorescence minus one (FMO) with the isotype antibody as the control), and applied to all samples. The total cell population was gated, and each EPC population with specific double-positive markers was analyzed using flow cytometry as described [14]. The fluorescence-positive cells were quantitatively evaluated using a Flow Cytometer LSRII (BD Bioscience, CA, USA). Cells positive for c-Kit^+^/CD31^+^, Sca-1^+^/Flk-1^+^, CD34^+^/CD133^+^, or CD34^+^/Flk-1^+^ were identified as EPCs. All antibodies were from Biolegend (SanDiego, CA, USA) except Flk-1 APC-Cy7, and CD34 FITC, which were obtained from Becton Dickinson Biosciences (Franklin Lakes, NJ, USA) and eBioscience (San Diego, CA, USA), respectively.

### 4.4. Intracellular ROS Measurement

Blood cells were harvested from mice after six weeks of PM or vehicle exposure for intracellular ROS analysis. RBCs were eliminated using RBC lysis, as described above [51]. The level of intracellular ROS formation in the blood cells was determined using the ROS Detection Reagent FITC (Invitrogen) as described [52]. After incubation with the reagent for 10 min at 37 °C, the labeled cells were washed twice with PBS, and then suspended in warm PBS for analysis using flow cytometry. The fluorescence-positive cells were quantitatively evaluated using an LSRII (BD Bioscience, San Jose, CA, USA) at a wavelength of 525 nm as described [51].

### 4.5. Cell Apoptosis Analysis

The apoptotic rate of circulating EPCs was determined using an FITC Annexin V apoptosis detection kit from BD (Cat#556547) according to the manufacturer’s protocol. Early apoptotic cells were defined as Annexin V-FITC-positive and propidium iodide (PI)-negative cells, and late apoptotic cells were defined as Annexin V-FITC and PI double-positive cells as described [53].

### 4.6. Ovariectomy

The ovariectomy surgery was performed as described [54]. Briefly, under general anesthesia (1%–2% isoflurane mixed with 100% oxygen), a 1 cm back skin incision was made with careful separation of the musculature. The bilateral ovarian fat pad was identified, and bilateral ovaries were gently exposed and removed surgically. The fat pad was then repositioned into the abdomen, and the wound was closed with separate muscle and skin layer closures. Similar procedures, but without removing the ovaries, were performed for sham control mice. Animals were placed on a heating pad throughout the procedure and recovery period. Mice were singly housed during recovery after surgery and monitored daily to ensure smooth recovery.

### 4.7. Statistical Analysis

All values are presented as mean ± standard error of the mean (SEM) and analyzed using one-way ANOVA (analysis of variance) followed by post-hoc conservative Tukey’s tests for three or more groups of data to minimize type I error as appropriate. Statistical analyses were performed using SPSS Statistics 18.0 and GraphPad Prism 8.4.2. Differences were considered statistically significant when *p* < 0.05.

## 5. Conclusions

The data from the present study demonstrated that PM exposure selectively decreases the number of circulating EPCs in male mice due to increased oxidative stress, an effect not noted in females. The protective effect on circulating EPCs in female mice against PM exposure appears to be mediated by an estrogen-independent mechanism.

## Data Availability

Upon reasonable request, the data are available from the corresponding author.
